# An exposure pathway‐based risk assessment system for GM plants

**DOI:** 10.1111/pbi.13146

**Published:** 2019-05-17

**Authors:** Yongbo Liu, Charles Neal Stewart

**Affiliations:** ^1^ State Key Laboratory of Environmental Criteria and Risk Assessment Chinese Research Academy of Environmental Sciences Beijing China; ^2^ Department of Plant Sciences University of Tennessee Knoxville TN USA

**Keywords:** exposure pathway, GM plants, risk assessment

Cultivating GM crops brings benefits, such as increasing farm incomes and the crop production as well as reducing insecticide and herbicide use and greenhouse gas emissions (Brookes and Barfoot, 2018). These benefits should translate into a brilliant future for GM crops. However, debates continue around GM food safety and environmental and ecological risk. Many GM opponents think scientists and agricultural corporations treat genes and genomes as resources to produce GM organisms (GMOs) at the expense of preserving nature and natural organisms (Dinneny, 2018). Nonetheless, ecological risk assessment of GMOs during pre‐ and post‐environmental release is very important to protect nature and provide consumers with safe crops. In order to address these broad goals, scientists and policymakers have proposed and adopted a range of ecological risk assessment approaches and systems.

Comparative risk assessment approaches are generally used to identify and evaluate differences between GM plants and their counterparts (Schnell *et al*., 2015). In addition to traditional toxicological methods, molecular profiling (e.g. ‘omics’ tools such as proteomics and metabolomics) has recently been added to comparative approach frameworks with the goal of increasing confidence in risk assessments of GMOs (Heinemann *et al*., 2011). Systematic review is considered as a powerful tool in informing evidence‐based risk assessment for GMOs, but it depends on sufficient and robust primary scientific data (Kohl *et al*., 2015). Risk–benefit analysis approaches have been proposed to serve as an additional support tool for decision makers (Morris, 2011). While food safety is a generalizable realm, ecological risk assessment is specific to regions and ecosystems. Policy articles stress specific ecological risks and strategies to change the focus of potential environments to real environments within a regulatory system and release region.

## Current ecological risk assessment systems

The Cartagena Protocol on Biosafety under the Convention on Biological Diversity (CBD) is an international agreement to guide the safe handling, transport and use of LMOs (living modified organisms, here using GMOs). Risk assessments undertaken pursuant to this Protocol focus on the potential effects of GMOs on the conservation and sustainable use of biodiversity in the receiving environment, taking also into account potential risks to human health. In 2016, the CBD proposed a guidance on risk assessment of LMOs (*UNEP/CBD/BS/COP‐MOP/8/8/Add.1*). The roadmap for risk assessment of LMOs includes overarching issues, planning phases (establishing the context and scope, problem formulation, choosing comparators), five steps of conducting the risk assessment (identification of novel genotypic and phenotypic characteristics, evaluation of likely adverse effects and their consequences, estimation of overall risk and recommendation). Some countries or inter‐country consorts have established frameworks on the risk assessment of GM plants for registration, such as the United States, EU and China (see the following paragraph).

At present, most frameworks aim to produce endpoint‐based risk assessments; that is the regulatory rules or statutes determine which endpoints need to be assessed (von Kries and Winter, 2012). Many risk assessment frameworks lack practical relevance to the actual environments where released plants will be grown. Risk assessments need to be more flexible to assure environmental relevance; the most important potential risks should be identified and tested on a case‐by‐case environmental basis. For example, in China, most criteria or guidance generally stipulate six risks to assess: survival and competition capacity of GM plants, gene flow from GM plants to their wild relatives, effectiveness of GM plants resisting target organisms, non‐target organism effects, community composition effects and pest evolution effects, and the risks of resistance evolution of target organisms (*Chinese Ministry of Agriculture. 2012. Guidelines for safety assessment of genetically modified plants*). In the United States, the EPA regulates GM plants according to biopesticide‐related statutes, which requires the assessment of non‐target organism effects, gene flow, resistance management and the fate of transgenic proteins in the receiving environment (*Introduction to Biotechnology Regulation for Pesticides*. https://www.epa.gov/). Moreover, the content of some assessment criteria or guidance has wasteful overlap and repetition. For example, one biosafety assessment technology criterion in China asks for the assessment of the effects of GM forest plants on non‐target organisms, pollinators and biological diversity simultaneously (*Chinese National Forest and Grassland Administration. 2007. Technical codes for biosafety assessment of genetically modified forest plants and products (LY/T‐1692)*). Sometimes, the requirements of risk assessment systems are not easy to follow. For example, the DIRECTIVE 2001/18/EC of the EU, ‘potential immediate and/or delayed environmental impact of the direct and indirect interactions between the GMO and target organisms’, in which the immediate and delayed environmental impacts are difficult for assessors to address.

In fact, most of the risk assessment systems are conducted according to potential consequences or proposed risks of GM plants in the receiving environment, based on transgenic characteristics (e.g. insect resistance and herbicide tolerance). The ‘case‐by‐case’ basis of risk assessment is accepted because risks depend on the organisms modified, a specific transgenic event and the receiving environment; this makes sense in many cases. However, this dogma may be difficult to execute in practice. The performance of relevant third‐party risk assessment could be technically and scientifically challenging, especially as traits and genetic engineering become more complex. Risk assessors need practical and detailed guidance to carry out their assessments. Thus, according to plant characteristics, we propose here an exposure pathway‐based risk assessment system, with fewer assessment phases but striving for holistic risk assessment.

## An exposure pathway‐based ecological risk assessment system

**Figure 1:**
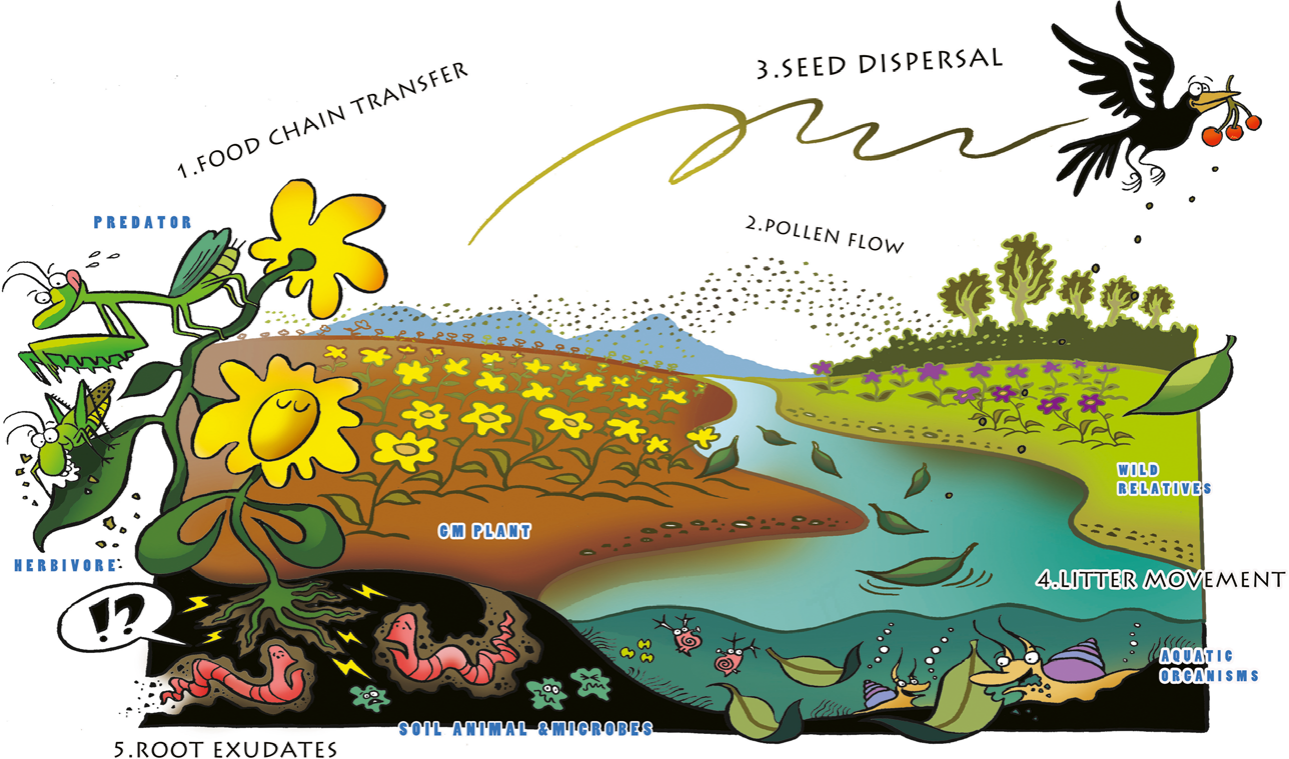
An exposure‐based risk assessment system for GM plants in actual receiving environments. The system includes five exposure pathways (food chain transfer, pollen flow, seed dispersal, litter movement and root exudates) in the environmental releasing of GM plants.

To improve the relevance, logic and comprehensiveness, we propose a risk assessment system that is based on exposure pathways for evaluating the ecological risks of releasing GM plants in specific environments. It does not consider laboratory testing of direct potential toxicity to non‐target organisms prior to environmental release, but rather focuses on the performance of GM plants in the receiving environment. The system includes five exposure pathways: pollen flow, seed dispersal, litter movement, root exudates and food chain transfer in the environment (Figure 1). To assess the ecological risks of GM plants in nature, we need only to test relevant hypotheses (problem formulation), measure or detect ‘exposure characteristics’ and potential ‘ecological effects’ for each exposure pathway. We propose integrating exposure characteristics and ecological effects to understand the ecological risks of GM plants in nature. The ‘ecological effects’ here are priori assumptions that are well‐trodden in scientific literatures and regulatory practice.

First, pollen flow by wind or pollinators may lead to the hybridization and introgression of GM plants with their wild relatives, depending on species and reproduction systems. The problem formulation of transgenic pollen flow is testing the hypothesis of producing hybrids through pollen‐mediated hybridization and, more importantly, genetic introgression. To evaluate the potential risk of transgenic pollen flow, we need to measure exposure characteristics: the distance of pollen flow, overlap days of flowering between GM plants and wild relatives, hybridization ratios and transgenic protein content. Then, measuring the ecological effects of pollen flow is needed: the hereditary stability and ecological adaptability of transgenic progeny. Each of these factors should be analysed to understand their contribution to stable genetic introgression, which is much rarer than hybridization (Stewart *et al*., 2003).

Second, the hypothesis of the dispersal of transgenic seeds through animals or human activities can lead to the presence of transgenic volunteers in nature. To evaluate the exposure characteristics of seed dispersal, we need to measure seed shattering of transgenic crops, the survival time of transgenic seeds in soil and the germination and dormancy of transgenic seeds relative to those of non‐transgenic crops. Evaluating the ecological effects of seed dispersal can simply detect the hereditary stability and ecological adaptability of transgenic volunteers. Through combining the exposure characteristics and ecological effects of seed dispersal, we could estimate the likelihood of transgenic volunteers persisting in the receiving environment.

Third, the movement of plant litter or residue through machines or human activities may lead to GM efflux into aquatic ecosystems. The hypotheses should address the ecological effect of exposure. To evaluate the exposure characteristics of litter movement, we should measure the movement distance and range of plant litter or residues, and the content of transgenic proteins in litters or residues. Evaluating the ecological effects of litter movement may be the changes in community composition (species and abundance) of aquatic organisms (e.g. plankton and benthos) relative to natural variation.

Fourth, transgenic proteins from root exudates of GM plants may be hypothesized to negatively affect soil and belowground microbiomes, that is a single‐tailed test. To evaluate the exposure characteristics of root exudates, we need to measure the distribution of plant roots, and the content of transgenic proteins in root exudates. Evaluating the ecological effects of root exudates can largely be accomplished by microbiome and community composition analysis, again, relative to natural variation.

Fifth, the problem formulation is hypothesizing that the transfer of transgenic proteins into food chains through insects feeding transgenic plants may directly or indirectly affect target and non‐target organisms. To evaluate the exposure characteristics of food chain transfer, we need to measure the content of transgenic proteins and the transfer ratio from one trophic level to the next one. Evaluating the ecological effects of food chain transfer should detect the evolution ratios of target organisms’ resistance to GM plants and the community composition (species and abundance) of non‐target organisms. Notably, direct or indirect toxicity to higher trophic levels would be required for risk to be found.

Our proposed holistic risk assessment system should be straightforward to test in a few model ecosystems to gauge ‘real‐world’ ecological risks of deploying GM plants using already‐approved GM plants. The results of exposure mode analysis can then be integrated to understand the ecological consequences in relevant environments. Based on the consequence estimations, the subsequent recommendations of whether to deploy, not deploy, or to deploy and mitigate could be rendered. While such a strategy may not completely alleviate every concern of GM opponents, it would provide relevant data for safety assurance. Indeed, in many cases GM technology improves the environment relative to growing non‐GM crops (Lu *et al*., 2012).

## Conflict of interest

The authors declare no conflicts of interest.
